# The Beneficiary Role of Selenium in Type II Diabetes: A Longitudinal Study

**DOI:** 10.7759/cureus.6443

**Published:** 2019-12-22

**Authors:** Dimitrios T Karalis

**Affiliations:** 1 Pathology, University of Thessaly, Volos, GRC

**Keywords:** type 2 diabetes, selenium, oxidative stress, hba1c, hdl, ldl, cholesterol, glucose, triglycerides, clinical trial

## Abstract

Introduction

Selenium (Se) is an antioxidotic element that is able to protect the pancreatic islets from oxidative stress, improve their functionality, and suspend atherosclerosis. The current paper is an attempt to demonstrate the beneficiary impact of administrating Se to patients with diabetes type 2 who are being treated with oral hypoglycemic agents, based on their glycemic and lipidemic profile.

Methods

The study involves 94 individuals, 72 male and 22 female patients aged 48 to 64 years old with diabetes mellitus type 2. They did not present any diabetic complications or significant comorbidities. They were following a Mediterranean diet and were monitored in order to maintain a steady body mass index (BMI). They were administered with Se 200 μg, taken once daily on an empty stomach. The laboratory testing included fasting blood glucose, hemoglobin A1c (HbA1c), total cholesterol, triglycerides, high-density lipoprotein (HDL), and low-density lipoprotein (LDL). The tests were performed before, three months after, and six months after the administration of selenium.

Results

The study resulted in a statistically significant reduction in the blood levels of glucose, HbA1c, cholesterol, and LDL in both three months and six months after the beginning of the treatment. HDL did not present any change during the first three months but did present a statistically significant increase in six months. Triglycerides did not present a significant reduction in both three and six months.

Conclusion

It appears that the administration of Se to type-2 diabetic patients can improve their glycemic and lipidemic profile, while larger definite trials are needed to provide further evidence.

## Introduction

Selenium (Se) is a mineral that is characterized as semi-metal or as a metalloid and is positioned in place 34 on the periodic table [1]. Se was discovered in 1818 by the Swedish Jons Jacob Berzelius; in 1957, Schwarz and Foltz identified it as being essential for human life [2]. Se exists in nature both in inorganic and organic form. In the organic form, Se is connected with proteins which are called selenoproteins [3]. The main organic forms are selenomethionine and selenocysteine [4].

Sources rich in Se are seafood, entrails, dairy products, grains, cereals and Brazilian nuts [5]. Se is a structural component of many enzymes such as glutathione hyperoxidase, Thioredoxin-Reduktase, deiodinase-Ιodothyronine and selenoprotein P [6]. Glutathione hyperoxidase includes selenocysteine [7] and constitutes a fundamental antioxidant component since it catalyzes the removal of organic hyper-oxides and hydrogen peroxide (Η_2_Ο_2_) from the tissues [8]. If these are taken out from the human body, they become toxic for cellular membranes and they will contribute to their destruction [7].

The present study attempts to propose Se as a dietary supplement (and not as a medicine) that can play a beneficiary role in type II diabetes. The participants were euglycemic and followed anti-diabetes treatment according to the protocols. Blood sugar was under control; therefore, our main goal was to enhance the therapeutic effect (interventional value of Se).

## Materials and methods

Hypothesis 

Oxidative stress has a huge impact on the etiology, pathogenesis, and the complication of type II diabetes. Due to its oxidative effect, glutathione hyperoxidase could play a crucial role against type II diabetes [9-10]. The excess of glutathione hyperoxidase in the pancreas could not only protect β-cells from oxidative stress but also improve their function. However, high dosages of Se could negatively affect the development of type II diabetes [9].

It is also hypothesized that antioxidants play an important role in fighting cardiovascular diseases by increasing the resistance of low-density lipoprotein (LDL) in oxidative modification. This boosts the formation of atherosclerotic plaques by regulating the synthesis of prostaglandin, accumulating blood cells, and protecting the organism against heavy metals (mercury) which have a toxic effect on the cardiovascular system [10-11]. The recommended average daily dosage of Se in adults is approximately 200 mg [12]. The present study hypothesizes that Se can improve the development of type II diabetes and risk factors of cardiovascular disease.

Methodology 

Prior to the main study, we have conducted a pilot study. The control group involved 40 participants. They were given a placebo. The results showed a non-statistically significant decrease in blood sugar and hemoglobin A1c (HbA1c). Total cholesterol, triglycerides, and low-density lipoprotein (LDL) cholesterol levels showed no change. High-density lipoprotein (HDL) cholesterol showed a small increase; however, it was not statistically significant. Both in the pilot study and in the main study, participants gave their written consent (according to the protocols of the National Ethics Committee for Clinical Studies).

In the main study, we have gathered 92 Greek patients, 70 males and 22 females, ranging from 48 to 64 years of age. All of them were smokers (approximately 20 cigarettes per day) and were diagnosed with type II diabetes. They were under medical surveillance, on anti-diabetic tablets, and they were relatively euglycemic. Their diet was under surveillance in accordance with the standards of the Mediterranean diet (carbohydrates: 50%, proteins: 18%-20%, fats: 32%-30%). They also had a stable daily intake of calories (2400-2600 kcal/day) so as to avoid any modifications in their body mass index (BMI). In the beginning of the study, their BMI was 30 and we have observed a relatively small decrease which was not statistically significant (work in progress to appear within 2019). The patients did not have any complications of type II diabetes and they did not have a record of other diseases. 

They were given 200 mg Se on a daily basis (without eating for four hours before taking the medication) for six months. The values of fasting glucose, HbA1c, blood total cholesterol, triglycerides, HDL, and LDL were measured within the time periods of zero, three, and six months after the beginning of the medication of 200 mg Se/day.

Fasting glucose was determined by the colorimetric method (normal values: 75-115 mg /d). HbA1c was determined by the high-performance liquid chromatography (HPLC) method (desirable percentage < 7%). Blood total cholesterol (normal values: 150-200 mg/dl) and triglycerides (normal values: 20-170 mg/dl) were measured by the enzymic colorimetry method.

## Results

The results were studied using the Statistical Package for the Social Sciences software (SPSS Inc., Chicago, IL). The distribution of differences in the dependent variable between the two groups was demonstrated normally since we were given a sample of n=92 (>50) participants.

**Figure 1:**
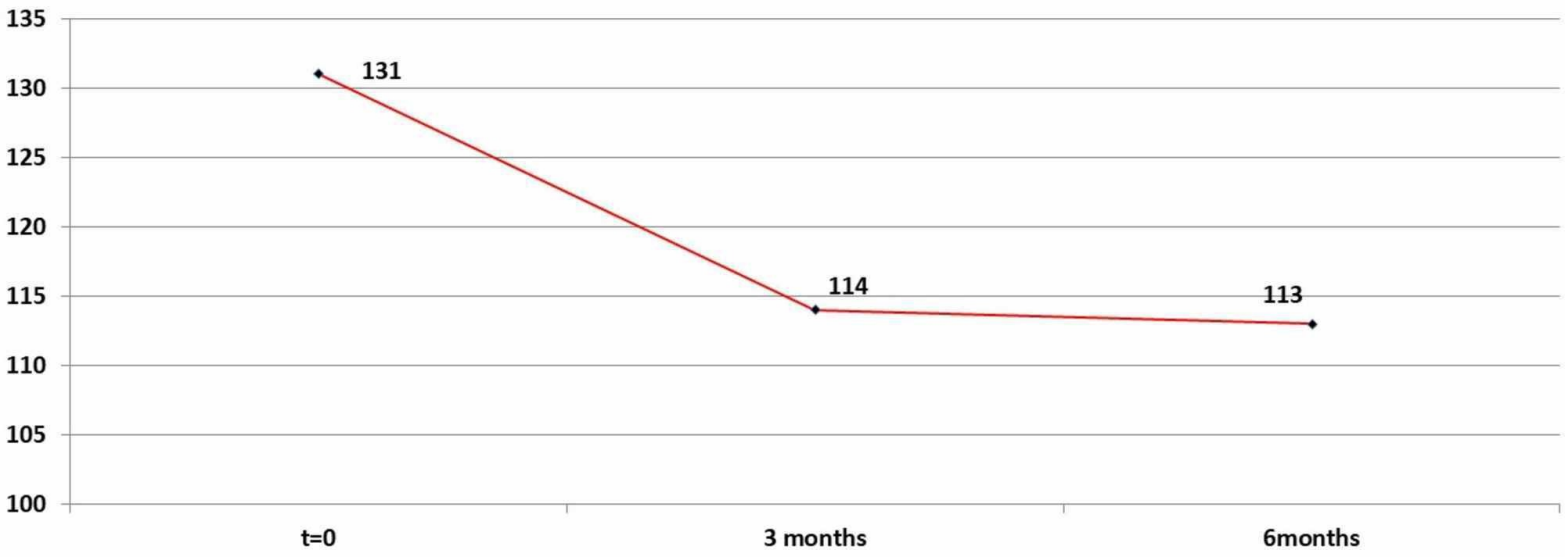
Average glucose levels

The administration of medication after three months lead to a significant decrease in blood glucose levels: 16,53 (mean=16,53). The results were statistically significant (p=0,00001<0,05). After six months of medication, the decrease of glucose was 17,21 (mean=17,21) compared to the day 0 (p=0,00001<0,5) as shown in Table 1 and Figure 1.

**Table 1:** Glucose descriptive statistics (zero, three, and six months)

	N	Range	Minimum	Maximum	Mean		Std. Deviation	Variance
					Value	Std. Error		
Glucose_Before	92	67	107	174	130,67	1,338	12,838	164,816
Glucose_after_3_Months	92	130,8	9,2	140	114,143	1,6307	15,6413	244,65
Glucose_after_6_Months	92	58	86	144	113,457	1,2334	11,8307	139,965
Valid N (listwise)	92							

**Figure 2:**
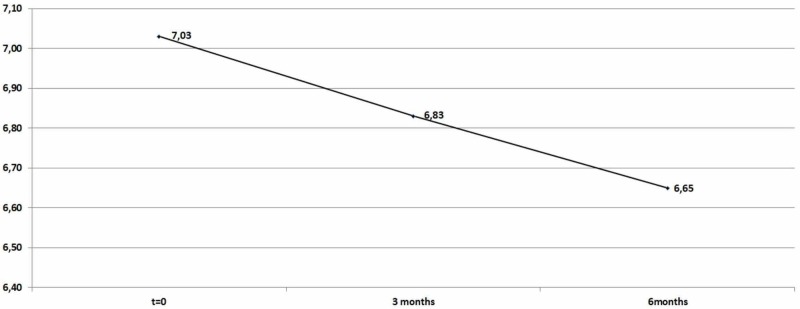
Average HbA1c levels HbA1c: hemoglobin A1c

The decrease in HbA1c levels was significant: 0,20652 (mean=0,20652) after the first trimester and 0,387 (mean=0,387) after six months as shown in Table 2 and Figure 2.

**Table 2:** HbA1c descriptive statistics (zero, three, and six months) HbA1c: hemoglobin A1c

	N	Minimum	Maximum	Mean	Std. Deviation
HbA1C	92	5,4	8	7,0326	0,4428
HbA1C_After_3_Months	92	5,9	7,6	6,8251	0,41027
HbA1C_After_6_Months	92	4,8	7,6	6,6499	0,55578
Valid N (listwise)	92				

**Figure 3:**
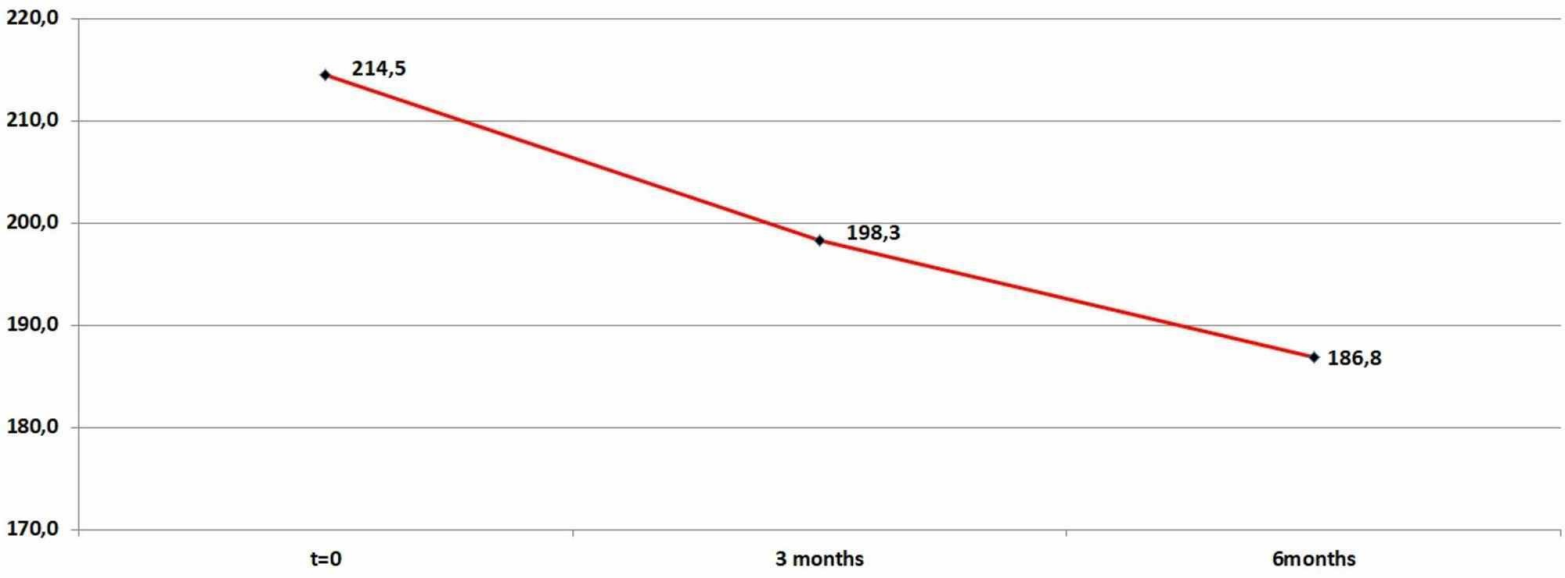
Average cholesterol levels

Moreover, the average value of blood total cholesterol ranged between 12,7463 and 19,7102 after three months of medication in Se and continued to drop after six months of medication (mean=27,7826). Therefore, the difference in blood total cholesterol levels as observed in the two time periods is highly significant as shown in Table 3 and Figure 3.

**Table 3:** Cholesterol descriptive statistics (zero, three, and six months)

	N	Minimum	Maximum	Mean	Std. Deviation
Cholesterol	92	178	254	214,543	18,5131
Cholesterol_After_3_Months	92	168	238	198,315	12,5365
Cholesterol_After_6_Months	92	165	224	186,751	10,9849
Valid N (listwise)	92				

**Figure 4:**
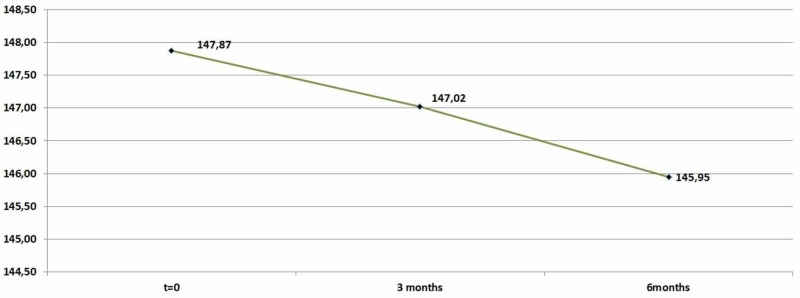
Average triglyceride levels

However, the results did not show a modification in triglyceride levels after three months of medication in Se (p-value = 0,660>0,05). However, triglyceride levels changed after six months of medication in Se compared to 0 time period (p- value=0,303>0,05). Therefore, meditation was not effective for this index as shown in Table 4 and Figure 4.

**Table 4:** Triglycerides descriptive statistics (zero, three, and six months)

	N	Minimum	Maximum	Mean	Std. Deviation
Triglycerides	92	88	187	147,8696	18,60357
Triglycerides_After_3_Months	92	107	182	147,0217	18,18282
Triglycerides_After_6_Months	92	104	174	145,9457	16,6029
Valid N (listwise)	92				

**Figure 5:**
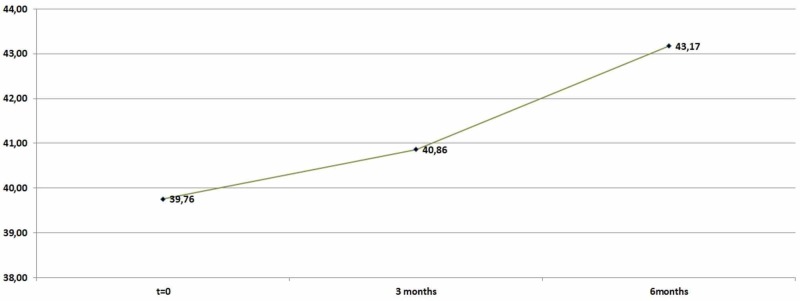
Average high-density lipoprotein (HDL) levels

As far as HDL levels are concerned, it could be ascertained (95%) that the average increase in HDL levels: 1,097 (mean- 1,097) was not statistically significant (p-value=0,098>0,05) for the first trimester. However, it was statistically significant (p=0,00001<0,05) after the time period of six months due to its increase (mean=3,4103) as shown in Table 5 and Figure 5.

**Table 5:** High-density lipoprotein (HDL) descriptive statistics (zero, three, and six months)

	N	Minimum	Maximum	Mean	Std. Deviation
HDL	92	22	54	39,7609	5,93809
HDL_After_3_Months	92	30	50	40,8587	4,94937
HDL_After_6_Months	92	31	56	43,1739	5,42199
Valid N (listwise)	92				

**Figure 6:**
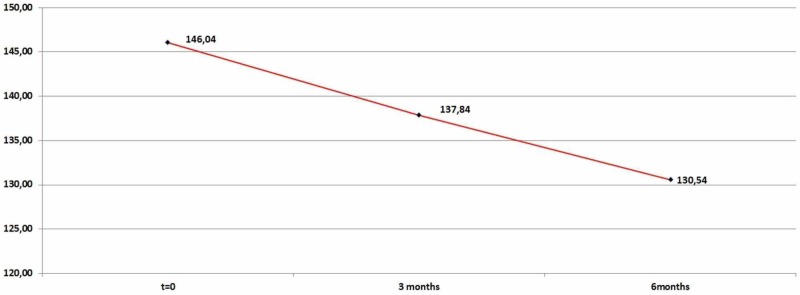
Average low-density lipoprotein (LDL) levels

Finally, LDL levels dropped significantly (p=0,0001) both for the time period of three months (mean=8,2065) and for the time period of six months (mean=15,50) as shown in Table 6 and Figure 6.

**Table 6:** Low-density lipoprotein (LDL) descriptive statistics (zero, three, and six months)

	N	Minimum	Maximum	Mean	Std. Deviation
LDL	92	110	198	146,0435	17,9345
LDL_After_3_Months	92	100	197	137,837	17,19397
LDL_After_6_Months	92	97	180	130,5435	17,4264
Valid N (listwise)	92				

## Discussion

There are not enough studies investigating the effect of Se in lipid profile and blood glucose. The present study showed that the administration of Se in the recommended dosages improves the values of fasting glucose, HbA1c, blood total cholesterol, HDL and LDL. However, there was not a significant difference in triglyceride levels. Different analyses of the Greek Healthcare System showed that increased (above average) Se levels in plasma relate with adverse lipid profile. 

Many studies stated that there was a correlation between dietary supplements and Se due to increased triglycerides and LDL levels. However, HDL levels dropped [13]. A study in diabetic patients showed that they may suffer from Se deficiency compared to non-diabetic patients [14]. A study conducted in France showed that elderly males with high Se levels had a lower risk of developing type II diabetes or having abnormal fasting glucose levels. Elderly females, however, did not run this risk at all [15]. 

In the USA, a study examined adults who were administered Se in the past. It was observed that HBA1C levels increased in proportion to Se levels [16-17]. Another longitudinal study examined Se levels in the nails of the big toes and showed that higher Se levels in nails relate to a lower risk of developing type II diabetes [18]. 

Lee et al. observed that individuals with low Se levels had an increased atherogenic index and lower HDL levels compared to individuals with higher Se levels. The correlation between Se levels and atherogenic index was tested only in females below 40 years of age [19]. 

Moreover, a study in Poland which was conducted in 2013 showed that Se supplements improved HBA1C levels but not fasting glucose levels [20]. However, a study conducted in Taiwan examined elderly individuals and found out that higher Se levels had a correlation with higher fasting glucose levels and with higher blood total cholesterol and triglyceride levels [21]. 

In 2016, a study on elderly individuals which was conducted in China ascertained that Se levels relate to type II diabetes due to increased glucose levels and their resistance to insulin [22]. Other Chinese scientists who carried out experimental studies during 2016 found out that increased Se levels relate with a more frequent presence of non-alcoholic fatty liver disease (NAFLD) and with increased triglyceride levels, LDL, fasting glucose, HBA1C, and insulin resistance [23]. 

Therefore, a comparison of the aforementioned findings suggests that there is a relative improvement after Se administration. However, there is no identical ground among the studies. Since there is a blank in the literature concerning the medicinal role of Se, the present research approaches it as a dietary supplement. It is suggested that as a dietary supplement, Se enhances the glycemic and lipidemic profile of type II diabetes patients because it can improve the effectiveness of their therapeutic treatment. It is likely that Se helps in delaying the development of pre-diabetes.

## Conclusions

The present study enforces the claims concerning the multiple benefits of Se as a dietary supplement in patients with type II diabetes under the prerequisite of following the Mediterranean diet as the recommended treatment method. Even though the present research is at an early stage, we could suggest that Se constitutes a necessary dietary supplement.
